# Could masked conceptual primes increase recollection? The subtleties of measuring recollection and familiarity in recognition memory

**DOI:** 10.1016/j.neuropsychologia.2012.07.029

**Published:** 2012-11

**Authors:** Jason R. Taylor, Richard N. Henson

**Affiliations:** MRC Cognition & Brain Sciences Unit, 15 Chaucer Road, Cambridge CB2 7EF, United Kingdom

**Keywords:** Remember/know, Source memory, Context, Episodic, Priming

## Abstract

We begin with a theoretical overview of the concepts of recollection and familiarity, focusing, in the spirit of this special issue, on the important contributions made by Andrew Mayes. In particular, we discuss the issue of when the generation of semantically-related information in response to a retrieval cue might be experienced as recollection rather than familiarity. We then report a series of experiments in which two different types of masked prime, presented immediately prior to the test cue in a recognition memory paradigm, produced opposite effects on Remember vs. Know judgments. More specifically, primes that were conceptually related to the test item increased the incidence of Remember judgments, though only when intermixed with repetition primes (which increased the incidence of Know judgments instead, as in prior studies). One possible explanation—that the fluency of retrieval of item–context associations can be experienced as recollection, even when the source of that fluency is unknown—is counter to conventional views of recollection and familiarity, though it was anticipated by Andrew in his writings nearly two decades ago.

## Highlights

► We review the estimation of recollection and familiarity, inspired by Andrew Mayes. ► In a recognition memory experiment, masked primes were shown before test cue words. ► Repetition primes increased ‘familiar’ responses, both hits and false alarms. ► Conceptual primes increased remember' responses (recollection), for hits only.

## 1 Introduction

We start with a brief review of theoretical and methodological issues relating to the estimation of recollection and familiarity within the recognition memory paradigm, before describing new data that would seem difficult to explain in terms of the dominant current conception of these two hypothetical processes. In the review, we focus on the contributions of Andrew Mayes, since few have considered these issues as deeply as has he. We then apply these theoretical and methodological considerations to new experiments using masked priming of test items, which produced a striking cross-over interaction between conceptual versus repetition primes and Remember/Know judgments.

### 1.1 A theoretical overview of recollection and familiarity in recognition memory

At its simplest, the recognition memory paradigm entails presenting a participant with a series of items (in a “study” phase), and then later (in a “test” phase), requiring them to make a two-way decision according to whether each of another series of items was, or was not, in the study phase. This paradigm has proved one of the most popular ways to investigate memory over the last few decades, most likely because of its simplicity, which is particularly helpful for patients with cognitive problems, for nonhuman animals, and for neuroimaging techniques.^1^ Whether this prevalence of the recognition memory paradigm is a good thing is a matter of debate – we suspect it has rather side-tracked the field (see also Hintzman, 2001) – nonetheless, the extensive data now accumulated from this paradigm deserve careful theoretical consideration.

Atkinson and Juola (1974) were probably the first to propose that participants can base their recognition decision on two, distinct retrieval processes; an idea that was extended by Mandler (1980), who introduced the terms recollection and familiarity. Similar ideas were developed by Humphreys (1978), Tulving (1985), Jacoby (1991), and others, leading to the popular “dual-process” model of recognition memory (see Yonelinas, 2002, for review). Since then, several variants of the basic recognition memory task have been used in attempts to separate the contributions of familiarity and recollection. The two main variants, which we consider in more detail below, are tests of Source Memory (which subsumes the Process Dissociation Procedure), and the Remember/Know procedure. However, interpretation of data from these procedures remains a deep and contested issue (e.g., see Migo, Mayes, & Montaldi, 2012).

First though, we need to introduce the conception of recollection and familiarity that we use in this paper. One common distinction between these concepts is in terms of subjective experience, or varieties of consciousness, as encapsulated, for example, by Tulving's (1985) Remember/Know procedure (see below). We prefer however to distinguish recollection and familiarity in mechanistic terms (similar to Greve, Donaldson, & van Rossum, 2010; Norman, 2010; and even Atkinson & Juola, 1974), derived from a detailed task analysis. As noted by others, recognition memory for familiar material (e.g., words) is really a test of contextual recall – not “have you seen this item ever before?” – but “did you see it in a specific context, i.e., the study phase?”. Despite this, the task can nonetheless be solved by an acontextual signal of memory strength, which might be proportional, for example, to the recency with which an item was last perceived. This is the sense of familiarity used here: a scalar signal, as might arise from a brain region involved in item perception/identification (e.g., perirhinal cortex, Brown & Xiang, 1998).

We assume that recollection, on the other hand, requires retrieval of (at least one bit of) new information from the study phase, that is not present at test. This is the same sense used by Montaldi and Mayes (2010): “…recollection is, therefore, a form of cued recall” (p. 1295)^2^ . The new information might be represented as a multidimensional pattern, for example, re-created over a distributed neural network (Norman & O'Reilly, 2003), in contrast with a unidimensional match signal derived from that network (Clark & Gronlund, 1996).

In order to be experienced as recollection, it is often assumed further that this retrieval of new information occurs within a dedicated episodic memory system (Schacter & Tulving, 1994), and/or during a special “retrieval mode” (Tulving, 1983, 1985). Familiarity, on the other hand, could reflect a match signal derived from episodic, semantic or some other (e.g., perceptual) type of memory system. Whether a specific recognition memory decision (such as a correct source judgment, or a remember judgment) corresponds to retrieval from episodic memory however, is not necessarily easy to infer (and runs the risk of circularity). Nonetheless, as expanded below, it is mechanistically possible to distinguish between direct retrieval of an episodic trace from episodic memory, versus retrieval from semantic memory of information associated with the test item, which can then be “matched” with one or more episodic traces.

#### 1.1.1 The subtleties of source memory tasks

**Fig. 1:**
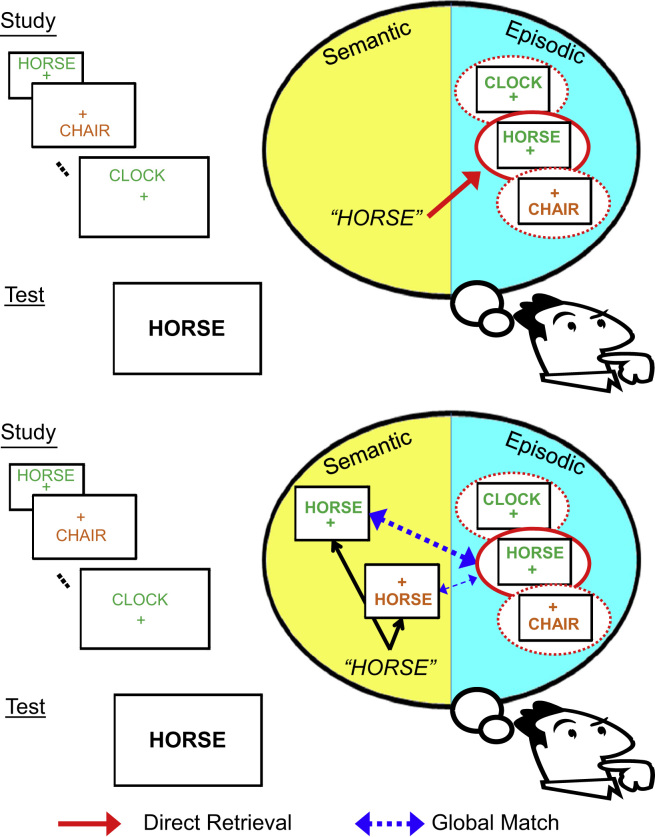
Schematic of an example source memory task, in which words are studied either above or below fixation, and either in green or orange. Two approaches to judging the source at test are direct episodic retrieval (recollection; upper panel) or a generate-and-recognise/global match strategy (familiarity; lower panel). See main text for further explanation.

Source information refers to the specific contextual information associated with prior exposure to an item that is targeted in a given task. Thus, in the study phase of a typical source memory experiment, participants might see each of a sequence of words displayed above or below a fixation point; then during test, words would be displayed in the centre of the screen, and participants would indicate not only whether they recognise the word from the study phase, but also (when they do recognise it) whether it was previously presented “high” or “low” on the screen (Fig. 1). Typically, participants decide between only a handful of source options (often just two) that are constant throughout the Test phase.

Various distinctions have been made between different types of source. Johnson, Hashtroudi, and Lindsay (1993), for example, distinguished between “external” source – viz information present in the environment at study, such as the location of a word on a screen – and “internal” source – viz information generated by the participant during study, such as which of two mental decisions was made about an item. A related distinction is whether the contextual information is bound as part of the representation of the studied item (“unitised”), such as the colour of an object, or represented separately, but associated with the target item, such as a detail about the room in which study took place. Some (e.g., Diana, Yonelinas, & Ranganath, 2010; Staresina & Davachi, 2008) suggest that retrieval of the former type of source (e.g., the colour of an object) might entail different mechanisms/brain regions, such as pattern completion in perirhinal cortex rather than hippocampus, than does retrieval of the latter (e.g., an object's location). Mayes, Montaldi, and Migo (2007), for example, distinguished associations between information represented within the same brain region, and associations between types of information represented in different brain regions. Here though, we regard retrieval of any source information from episodic memory as recollection, regardless whether that source is internal/external, unitised/non-unitised, or within/between information-type.

There are at least two well-known caveats with the typical source memory paradigm. The first is the problem of guessing: If there is only a small number of distinct source-types, and one is interested in differences associated with correct versus incorrect source decisions (e.g., in terms of reaction times, ERP amplitude, or BOLD amplitude), then a significant proportion of those decisions are likely to be guesses. One simple solution is to provide a third response option for guesses (e.g., Staresina & Davachi, 2008), though more generally, one would obtain a concurrent confidence judgment associated with each source decision, which provides greater latitude to separate recollection from confidence (e.g., Mickes, Wais, & Wixted, 2009; Slotnick, 2010).

A second caveat, particularly relevant to neuroimaging analyses (see, e.g., Montaldi & Mayes, 2011), is that an incorrect source decision does not imply that no recollection has occurred. This is simply because, though a participant may not recall the type of source targeted by the task (e.g., location of the item on a screen), they might recall some other contextual detail. This issue of “non-criterial recollection” (Yonelinas & Jacoby, 1996) has been well-known for many years. However, what has been less often appreciated is that, even when a source decision is correct (and not simply a guess, e.g., given with high confidence), this need not imply recollection has occurred, as expanded below.

If one assumes separate semantic and episodic memory systems, then a further problem with the source memory paradigm arises if participants are able to generate associations of the test item (from their semantic memory), and then match those associations with each episodic trace (in their episodic memory). Such a “generate-and-recognise” process (Watkins & Gardiner, 1979) would not correspond to recollection, in the present sense of recall (retrieval of additional information) from episodic memory. Rather, this retrieval from semantic memory, followed by a global match with each trace in episodic memory, would correspond to an assessment of familiarity.

This potential distinction is illustrated in Fig. 1. The first possibility, shown in the top panel, is that the cue provided by the test item directly retrieves a specific trace from episodic memory, which allows the source decision to be made, i.e., recollection. An alternative possibility however, shown in the bottom panel, is that, being aware that there are only two locations and two font colours, the participant imagines the cue word either above or below a fixation cross (i.e., constructs a visual image based on their semantic knowledge), and either in green or orange; i.e., generates up to four images in this case. One of these images may then result in a sufficient match against the traces in episodic memory that a sense of familiarity with that image is experienced, allowing the participant to effect a correct source decision. This process does not entail recall of additional information from episodic memory, but might correspond to a type of associative familiarity (Yonelinas, 1997).

This generate-and-recognise strategy is feasible if only a few source options are probed in the experiment. This would appear to relate to the concern of Montaldi and Mayes (2010), when they note: “Participants may hold these two or three sources in their minds throughout the task, so that success does not indicate recollection (i.e., cued recall), but associative familiarity” (p. 1303). It is less clear however that “this problem can only be effectively addressed by having unique item–source pairings with adequate numbers of items” (Montaldi & Mayes, 2010, p. 1303). Having unique item–source pairings would seem neither necessary nor sufficient. What would appear to matter is the number of response options (source attributes) associated with each test item: A small number of options (even if varied each trial) is always going to invite participants to generate combinations of the test item with each source option, and see if any combinations seem familiar. In other words, the best way to avoid this problem would be to have a large (open-ended) response set: i.e., require recall of any of a large number of source attributes. Indeed, the same problem of generate-and-recognise strategies applies to the process dissociation procedure (Jacoby, 1991), when participants must exclude test items on the basis of a single, specific source attribute. It can also apply to associative recognition paradigms (where the task is to distinguish intact from re-arranged pairings of studied items; Yonelinas, 1997), if one item in each pair can be generated from the other, e.g., if semantically related.

Of course, this hypothetical relationship between generate-and-recognise strategies, familiarity and recollection makes certain assumptions (e.g., that distinct episodic and semantic memory systems exist), and is difficult to test in practice. In Fig. 1, for example, one can also imagine an intermediate case, where a participant imagines the test word in its correct location at study (but not in any specific colour), and this cue is sufficient to retrieve an episodic trace, now complete with the studied colour too. This would be an example of partial recollection: Where part of the source was generated from semantic memory (the location), allowing retrieval of another aspect of source from episodic memory (the colour). We return to this possibility when discussing our present data.

#### 1.1.2 The subtleties of the Remember/Know procedure

The Remember/Know (R/K) paradigm is often assumed to be a direct way of assessing recollection and familiarity, simply by asking participants to introspect on their recognition decision. The paradigm was originally introduced by Tulving (1985), in an attempt to assess whether retrieval has occurred from episodic or semantic memory systems. According to Tulving, “remembering” reflects conscious memory of oneself in the past (“autonoetic” consciousness, or “mental time travel”), while “knowing” reflects an experience associated with the past in the absence of such self-memory (“noetic” consciousness). Since then, numerous dissociations between R/K judgments as a function of various experimental manipulations have been reported (see Gardiner, Ramponi, & Richardson-Klavehn, 2002, for review), and these have been interpreted in various ways (e.g., Gardiner & Parkin, 1990; Rajaram, 1993; Jacoby et al., 1997), the most popular of which is in terms of independent processes of recollection and familiarity (Yonelinas & Jacoby, 1995). Nonetheless, the relationship between the empirical labels of R and K and the underlying hypothetical constructs of recollection and familiarity – for example, whether that relationship is exclusive, independent or redundant – remains a matter of debate (e.g., Knowlton & Squire, 1995), as recently reviewed by Mayes et al. (2007). Furthermore, others have argued that the empirical dissociations between R/K judgments can be explained more simply in terms of signal-detection theory (SDT), as different response criteria placed on a single continuum of memory evidence (e.g., Donaldson, 1996; though multiple, qualitatively different contributions to that evidence are not ruled out by a unidimensional SDT model of the decision itself; Wixted & Mickes, 2010; and there are also multidimensional SDT approaches, e.g., Rotello, Macmillan, & Reeder, 2004).

Despite these unresolved issues, and the attractiveness of the alternative source memory paradigm in providing an objective rather than purely subjective measure, we have preferred the R/K paradigm, mostly because of the other difficulties with the source memory paradigm raised in the previous section. Foremost, by not being restricted to a specific source attribute, R judgments should be an exhaustive measure of recollection (cf. non-criterial recollection in source memory paradigms). Furthermore, by not focusing the participant on a specific type of source, it would seem less likely that participants will spontaneously adopt a generate-and-recognise strategy (e.g., based on what they expect to be the common types of source). In other words, they are less likely to adopt a specific retrieval orientation (Rugg & Wilding, 2000). Indeed, such strategies are further discouraged in the “modified R/K” procedure developed by Mayes, Montaldi and colleagues (Mayes et al., 2007), in which attempts to recollect are discouraged, such that R judgments are reserved only for incidental recollections.

However, the above argument about exhaustiveness of R judgments is, of course, predicated on the participant fully understanding and complying with what is meant by “remembering”—viz, retrieving at least one bit of information that is not present at Test, nor generated from some other source such as semantic memory. Foremost, this assumes that participants can tell whether additional information that comes to mind has come from their episodic memory, rather than from, say, semantic memory (Tulving, 1983). One simple solution is to assume that information retrieved from episodic memory contains a unique episodic “tag” (e.g., some unique spatiotemporal contextual information). Another possibility however relates to the unexpected (and/or unintended) nature or fluency of that retrieval; an issue to which we return in the General Discussion in terms of attributing the ease of retrieval to recollection.

Furthermore, in practice, participants may experience some trials when they retrieve many contextual details, and other trials in which they only retrieve one, and hence decide to impose their own criterion for giving an R judgment. Indeed, this can be modelled by assuming the amount of recollected information represents a continuum (Rotello et al., 2004; Wixted & Mickes, 2010). In this case, an absence of an R judgment does not mean that no recollection occurred (again, with implications for any concurrent neuroimaging data). However, this emphasises the importance of the R/K instructions given to participants: We would emphasise that retrieval of just one contextual detail is sufficient to justify an R judgment, even if this would mean that participants are less confident of some R judgments than others (when multiple details are retrieved).

The importance of precise R/K instructions is further reinforced by demonstrations that different results are obtained depending on: (i) whether R/K instructions do, or do not, emphasise that remembering and knowing are not the same as high and low confidence (Geraci, McCabe, & Guillory, 2009); (ii) whether an additional “guess” option is included (Gardiner et al., 2002); (iii) whether R/K judgments are made after the initial old–new judgment (a two-step procedure), or concurrently (in a R/K/new, one-step procedure; Hicks & Marsh, 1999); (iv) as a function of the specific response labels used (e.g., McCabe & Geraci, 2009, who found more accurate use of these concepts when they were labelled “type A memory” and “type B memory”, to avoid colloquial meanings associated with “remember” and “know”); and (v) whether exclusive or parallel R/K ratings are used (Higham & Vokey, 2004). In the experiments below, we address most of these concerns, using a two-step procedure, with instructions based on Rajaram (1993) that emphasise the difference between remembering, knowing, and confidence, and use the term “familiar” rather than “know”, to minimise colloquial biases associated with “knowing” (though we retain the labels “R” and “K” here for consistency with the literature). Even if these precautions are insufficient (e.g., if interpretation of “familiar” suffers from the opposite problem as “knowing”, in suggesting lower confidence), the cross-over dissociation that we find between R/K judgments and type of masked prime would seem difficult to explain solely in terms of a conflation with confidence/guessing. However, the use of parallel rather than exclusive ratings of R and K (e.g., Brown & Bodner, 2011; Kurilla & Westerman, 2008), in terms of the implications for participants’ interpretation of recollection and familiarity, is an important issue to which we return in the Discussion to Experiment 1.

### 1.2 A new dissociation in Remember/Know judgments produced by masked primes at test

Having reviewed the pros and cons of common methods to assess recollection within the recognition memory paradigm, we turn now to the specific background associated with the present experiments. One variant of the recognition memory paradigm that has been used to support dissociable contributions from recollection and familiarity was originally introduced by Jacoby and Whitehouse (1989). In this variant, each test item is preceded by a brief, masked prime, which either does, or does not, match the test item (e.g., is either the same or a different word). When participants were not aware of the prime, Jacoby and Whitehouse found participants were more likely to report the test item as studied when it was preceded by a matching than non-matching prime, regardless of whether or not the test item was in fact studied. The authors attributed this “memory illusion” to an increased fluency of processing test items that were preceded by matching primes: In the absence of awareness for the true source of that fluency (i.e., the primes), they proposed that participants erroneously attribute that fluency to prior processing in the study phase. This explanation sits easily with a dual-process interpretation that masked primes affect familiarity, rather than recollection. Indeed, a subsequent study by Rajaram (1993) used the “standard” R/K procedure, and confirmed that the masked repetition primes increased K judgments (to both studied and unstudied items), but not R judgments (see also Woollams, Taylor, Karayanidis, & Henson, 2008; though see Lucas, Taylor, Henson, & Paller, this issue; Tunney & Fernie, 2007).

This effect of repetition priming on familiarity is often interpreted as due to *perceptual* fluency. However, because two identical words (in different case) are similar in terms of perceptual features, and identical lexically and conceptually, it is unclear whether the locus of the effect is truly perceptual. A study by Rajaram and Geraci (2000) used semantic primes, and found the same priming-related increase in K but not R judgments that has been found using repetition primes. This result suggests that the familiarity signal arises at the level of conceptual fluency (consistent with Yonelinas, 2002). However, because Rajaram and Geraci's primes were also associatively related to the test (target) item, it is possible that any increase in familiarity was due to lexical rather than conceptual fluency: Although many associated words are also conceptually related, associative probability is influenced by non-conceptual factors such as the probability of co-occurrence in language (e.g., hobby-HORSE, grand-PIANO), and in semantic priming studies, association tends to dominate over conceptual relatedness (Lucas, 2000). Furthermore, the primes in Rajarm and Geraci's procedure were unmasked (clearly visible), which has previously been found to eliminate, or even reverse, repetition priming effects on recognition memory (e.g., Jacoby & Whitehouse, 1989; see also Higham & Vokey, 2004; Klinger, 2001).

In the following experiments, we attempted to isolate the effect of conceptual fluency on Remember/Know judgments by using primes that shared semantic attributes, but were not associatively related to the test cue (e.g., cow–HORSE; guitar–PIANO), and which were sandwich masked to reduce participant's awareness of them. In Experiment 1, the effects of these masked, conceptual primes on recognition decisions to a subsequent target word were compared with those of repetition primes (in relation to unprimed trials, where the prime word was unrelated to the target word). In Experiments 2a and 2b, we explored the effects of these conceptual primes without concurrent exposure to repetition primes.

## 2 Experiment 1

### 2.1 Materials and methods

#### 2.1.1 Participants

Participants were recruited from the student population of Cambridge University; all participants had normal or corrected to normal vision and were right-handed (self-report). Experiments were of the type approved by a local research ethics committee (LREC reference 05/Q0108/401). A total of 26 participants (12 females) gave informed consent to participate, with a mean age of 20 (*SD*=2) years.

#### 2.1.2 Materials

The stimuli consisted of 480 word-pairs (“prime”–“TARGET”) that were conceptually related but not lexically associated according to word-generation norms (both forward and backward association probabilities <.10 in the USF norms; Nelson, McEvoy, & Schreiber, 1998). Conceptual relatedness was defined on the basis of taxonomic category (e.g., piano–GUITAR, horse–COW), attributes or functions (e.g., silver-COIN, teapot-BOIL), typical context (e.g., pond-FROG, wedding-BRIDE), part–whole relationship (e.g., tobacco-CIGAR, camera-LENS), or lexical interchangeability (e.g., biscuit-COOKIE, shop-BOUTIQUE). All primes and targets were between 3 and 8 letters long (primes: *M*=5.26, *SD*=1.12; targets *M*=5.44, *SD*=1.38) and had written frequencies between 1 and 150 per million (primes: *M*=33.97, *SD*=26.00; targets *M*=34.14, *SD*=36.08; Kucera & Francis, 1967). These conceptually related prime–target pairs comprised the Primed condition in Conceptual Priming blocks; two further lists were created by re-pairing each target with itself (Primed condition, Repetition Priming blocks) or with an unrelated prime via a pseudo-random shuffle (Unprimed conditions in both Conceptual and Repetition Priming blocks). These lists were each further sub-divided into four Sets (A–D) for the counterbalancing described below.

#### 2.1.3 Procedure

The experiment consisted of four cycles of Study-Test blocks. During each Study block (60 trials+4 “dummy” trials, 2 at the beginning and 2 at the end, to prevent primacy and recency effects, ignored in analysis; approximately 2.5 min), participants performed an “interestingness” judgment (based on our previous studies, e.g., Taylor, Buratto, & Henson, in press; Woollams et al., 2008): They read each word and indicated whether it was *interesting* or *not* by pressing one of two buttons. During each Test phase (120 trials+2 “practice” trials at beginning, ignored in analysis; approximately 10 min), participants performed a yes/no recognition task, in which they first indicated whether they thought each test item had (*old*) or had not (*new*) appeared in the previous Study phase. If they responded “old”, they were also prompted to decide whether they *remembered* seeing the test item (“R” judgment) or whether the item was *familiar* (“K” judgment). Note that we used the label “familiar”, rather than the traditional “know” judgment, for reasons given in Section 1.1.2. Participants were trained in this distinction using instructions based on Rajaram (1993; see Taylor et al., in press) and prior training (16 practice test trials). If the participant responded “new” to the test cue, or if they failed to respond (time limit=2000 ms), the procedure continued to the next trial (on the rare occasions where no response was given, a “new” response was assumed).

**Fig. 2:**
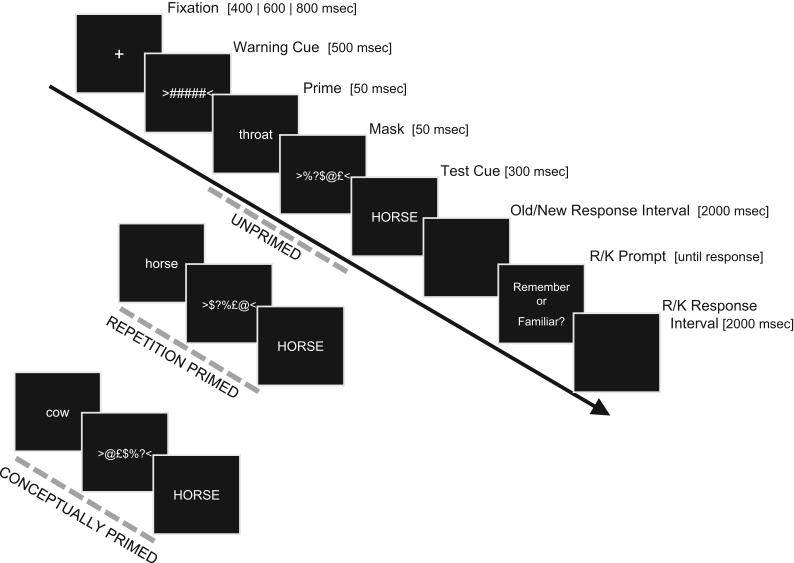
Schematic of trial procedure at Test, and examples of each of the three types of Prime (Conceptual, Repetition or Unrelated). Duration of each event is given in square brackets (note that spacing of objects on the timeline does not reflect duration).

On Test trials, the test item (“target”) was preceded by a brief, masked prime word (see Fig. 2 for trial timing). In Conceptual Priming blocks (either the first two or last two Test phases; order counterbalanced across participants), prime–target pairs were either conceptually related (Primed trials, 50%=60 trials/block) or unrelated (Unprimed trials, 50%); in Repetition Priming blocks, prime–target pairs were either the same word (Primed trials, 50%) or unrelated words (Unprimed, 50%). Primes were presented in lower case and targets in upper case, to minimise visual overlap on Repetition priming trials. No word was repeated across blocks.

Block Order (Repetition/Conceptual Priming first) and Set-Condition mapping (A/B/C/D→Repetition/Conceptual×Primed/Unprimed) were counterbalanced across participants, with a total cycle of eight participants. Stimuli were presented on an LCD computer screen (60 Hz refresh rate) positioned approximately 70 cm in front of the participant. Words were presented in white on a black background. Responses were made with right and left index fingers, with response mappings counterbalanced separately across Interestingness, Old/New, and R/K decisions. Stimulus presentation and response collection was performed using E-Prime 1.0 (Psychology Software Tools).

Upon completion of the main experiment, subjective and objective measures of prime awareness/visibility were collected. First, participants were asked whether they noticed any “hidden words” (i.e., the masked primes) in the procedure, and whether they had been able to identify any of these words (subjective measures). The nature of the experiment, and in particular of the masked primes, was then explained, and participants performed a Prime Visibility Test. In this test, 120 test trials were shown as during the experiment (fixation, forward mask, prime, backward mask, test cue), and participants were asked to indicate which of three (equally likely to be correct across trials) candidate words had been the prime on that trial (i.e., 3AFC). The three candidate primes were (a) the same word as the target (i.e., a Repetition prime), (b) a conceptually related word (i.e., a Conceptual prime), and (c) an unrelated word (Unprimed condition). Participants were instructed to guess if they were unsure, and the next trial began only after a response was made.

#### 2.1.4 Data analyses

The data (proportion of trials and, separately, response times) were analysed in multifactorial repeated-measures analysis of variance (ANOVA). Of particular interest were effects involving the Prime Status factor, i.e., priming effects; effects not involving Prime Status are reported only when relevant. Significant effects are only reported in the absence of significant higher-order interactions. All statistical tests had alpha set at .05, and a Greenhouse–Geisser correction was applied to all *F*-values with more than one degree of freedom in the numerator. *T*-tests were two-tailed, except where stated otherwise.

### 2.2 Results

#### 2.2.1 Proportion of trials

The mean proportions of responses in each condition are shown in Table 1. For R judgments, overall accuracy (Pr, the proportion of Hits minus the proportion of False Alarms, averaged across primed vs. unprimed) was .57 in Conceptual Priming and .59 in Repetition Priming blocks, both significantly greater than zero, *t*(25)s>10.0, *p*s<.001. For K judgments, familiarity was calculated under independence assumptions (“IRK” procedure; Yonelinas & Jacoby, 1995), where familiarity=*K*/(*N*−*R*); *R*=number of R judgments; *K*=number of K judgments and *N*=total number of test trials (non-independent scores are reported in footnotes below). Familiarity Pr values were then .52 in Conceptual Priming and .49 in Repetition Priming blocks, both of which were significantly greater than zero, *t*(25)s>10.0, *p*s<.001.^3^

**Fig. 3:**
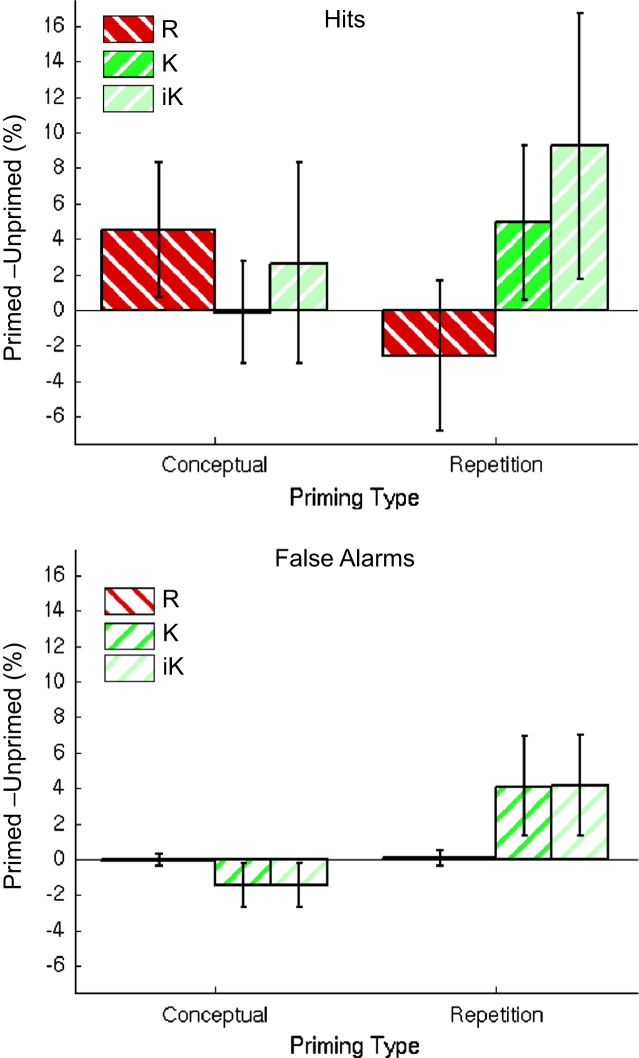
Priming (% of trials called “old” for Primed–Unprimed) as a function of Studied (Hits; upper panel) or Unstudied (False Alarms; lower panel) items, for Conceptual vs. Repetition Primes and R (red) vs. K (green) judgments in Experiment 1. The two bars for K judgments are based on raw numbers (dark green) or under independent (IRK) scoring (‘iK’, light green; see main text). Error bars are 95% confidence intervals.

For factorial ANOVA analyses, separate recollection and familiarity IRK estimates were made for studied (i.e., hits) and unstudied (i.e., correct rejection) trials, and for each priming condition. For “old” judgments, the 2 (Priming Type)×2 (Memory Judgment)×2 (Study Status)×2 (Prime Status) ANOVA revealed a significant 3-way interaction between Priming Type×Memory Judgment×Prime Status, *F*(1,25)=7.87, *p*<.01, which indicated that the pattern of R- and K-priming effects differed between Conceptual and Repetition Priming blocks. The nature of this interaction is apparent in Fig. 3, which shows Priming Scores (Primed–Unprimed) as a function of R/K judgment and Priming Type, separately for Hits (Studied words) and False Alarms (Unstudied words; error bars are 95% confidence intervals; raw and “independence” K shown separately). Conceptual priming caused a significant increase in R (but not K) judgments; Repetition priming, on the other hand, caused a significant increase in K (but not R) judgments.

Even though no interaction involving Study Status and Prime Status reached significance under IRK scoring (unlike exclusive scoring, under which the Priming Type×Study Status×Prime Status was significant; see footnote 3), the confidence intervals in Fig. 3 show that the Conceptual priming effect on R was only significant for Studied items (Hits), whereas the Repetition priming effect on K occurred for both Studied and Unstudied items (False Alarms).

### 2.3 Response time

Response times (RTs; medians) for correct “old” (Hit) and “new” (CR) decisions (there were too few False Alarms and Misses to include these), shown in Table 2, were analysed in a 2×3×2 ANOVA with factors Priming Type (Conceptual, Repetition), Memory Judgment (R, K, CR), and Prime Status (Primed, Unprimed). Two participants were excluded because they had insufficient number of trials (<2) in at least one cell of the design.

The ANOVA revealed a main effect of Memory Judgment, *F*(1.88,43.3)=38.8, *p*<.001, and follow-up *t*-tests showed that RTs to correct “old” decisions subsequently given an R judgment (*M*=836 ms, *SD*=93) were significantly faster than those subsequently given a K judgment (*M*=1012 ms, *SD*=138), *t*(23)=9.28, *p*<.001. CRs were also faster than Ks, *t*(23)=6.85, *p*<.001; R and CR RTs did not differ (*p*>.1). A main effect of Prime Status was also found, *F*(1,23)=5.55, *p*<.05, indicating that participants were faster to respond in the Primed (*M*=890 ms, *SD*=104) than in the Unprimed condition (*M*=913 ms, *SD*=98). Additionally, the interaction between Memory Judgment and Prime Status was significant, *F*(1.62,37.2)=6.53, *p*<.01; follow-up *t*-tests showed that the priming effect (Unprimed–Primed, collapsed across Conceptual and Repetition blocks) was significantly larger for R (*M*=29 ms) than for CR (*M*=−9 ms), *t*(24)=3.24, *p*<.01, and significantly larger for K (*M*=48 ms) than for CR, *t*(23)=3.09, *p*<.01.

### 2.4 Prime visibility test

In our subjective test of prime visibility, 16 of the 26 participants (62%) reported being aware that there were “hidden” words in the experiment; 6 of these “aware” participants reported being able to identify prime words on some trials. In our objective test of prime visibility, overall accuracy was 68.2% (*SD*=24.0%), which was significantly better than chance (33%), *t*(25)=7.46, *p*<.001. Though mean accuracy was greatest for Conceptual primes (*M*=74.7%, SD=26.0%), intermediate for Repetition Primes (*M*=67.2%, SD=33.6%) and least for Unrelated primes (*M*=62.6%, SD=32.4%), there was no significant effect of Prime Type in a one-way ANOVA, *F*(1.60,40.1)=1.76, *p=*.19.

**Fig. 4:**
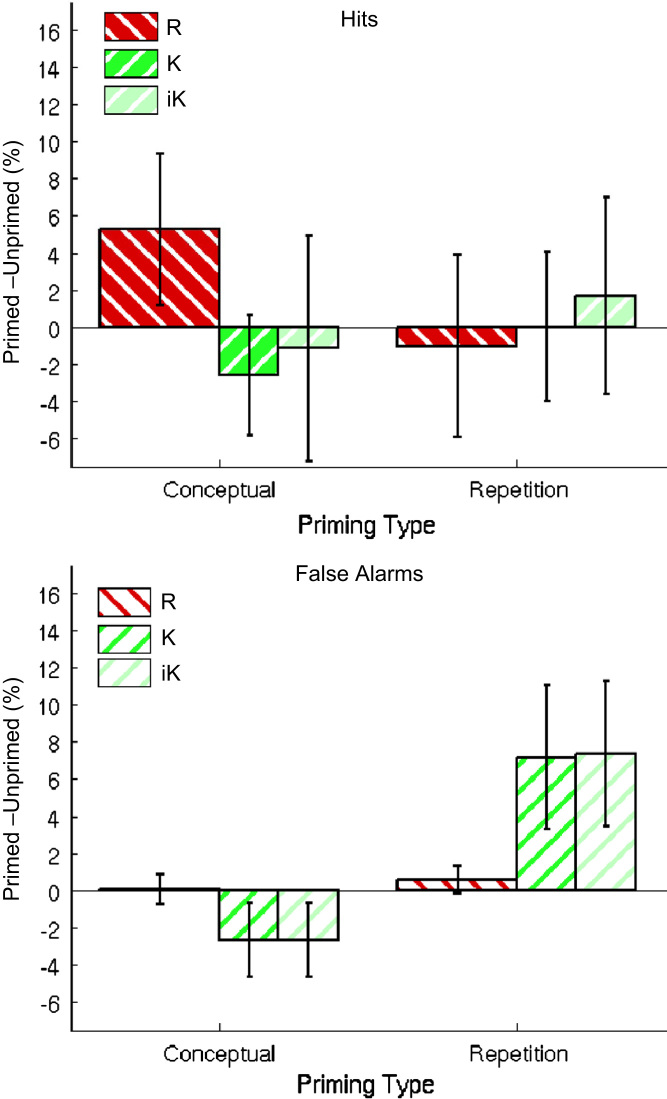
Priming effects for participants who reported being unaware of primes, combining data from Experiment 1 with data from a different group of participants reported in Taylor et al. (in press). Priming (% of trials called “old” for Primed–Unprimed) is shown as a function of Studied (Hits; upper panel) or Unstudied (False Alarms; lower panel) items, for Conceptual vs. Repetition Primes and R (red) vs. K (green) judgments in Experiment 1. The two bars for K judgments are based on raw numbers (dark green) or under independent (IRK) scoring (‘iK’, light green; see main text). Error bars are 95% confidence intervals.

Given that awareness of the primes might have affected the present results – e.g., the increased tendency to give R judgments following conceptual primes – we repeated the above ANOVA on proportions of “old” judgments, but with an additional between-participant factor of aware vs. unaware. To increase the power of this analysis, we added data from the 22 participants who performed the identical paradigm within an fMRI scanner (reported in Taylor et al., in press), of whom 9 reported awareness (resulting in *n*=25 in the “aware” group, and *n*=23 in the “unaware” group). The three-way interactions of Priming Type×Memory Judgment×Prime Status, *F*(1,46)=7.1, *p*<.05, and of Priming Type×Study Status×Prime Status, *F*(1,46)=8.7, *p*<.01, remained the highest order significant interactions, as in the previous analysis.^4^ Importantly, the group factor of Awareness did not interact with either of these effects, *F*s<2.2, *p*s>.14, or indeed any other effect, *F*s<2.52, *p*s>.11. Furthermore, when the main ANOVA was performed on the *n*=23 “unaware” participants only, the same three-way interactions of Priming Type×Memory Judgment×Prime Status, *F*(1,22)=5.5, *p*<.05, and of Priming Type×Study Status×Prime Status, *F*(1,22)=5.8, *p*<.05, remained the highest order significant interactions, as in the previous analyses.^5^ Indeed, the pattern of significant priming effects matched that from all participants from Experiment 1: being significant only for R Hits following conceptual primes, and K False Alarms following repetition primes, though no longer quite reached significance for K Hits following repetition primes (Fig. 4).

Finally, given possible limitations in our subjective measure of prime awareness (e.g., in reluctance or incompleteness of some participants’ self-report^6^), we also looked for correlations across all *N*=48 participants between their amount of priming and their objective prime visibility accuracy. There was no sign of a positive correlation for Conceptual primes followed by R judgments (*r*=−.22, *p*=.12, even if restricted to R Hits, *r*=−.23, *p*=.11), or followed by K judgments (*r*=.16, *p*=.12, even if restricted to K FAs, *r*=−.01, *p*=.94; also non-significant for raw K judgments); nor for Repetition primes followed by R judgments (*r*=−.03, *p*=.81, even if restricted to R Hits, *r*=−.02, *p*=.87), or followed by K judgments (*r*=.03, *p*=.39, even if restricted to K FAs, *r*=.01, *p*=.97; also non-significant for raw K judgments). Thus we found no evidence that the present pattern of priming effects, particularly the interaction between Conceptual vs. Repetition primes and R vs. K judgments, was affected by how likely the participants were to have seen the primes.

## 3 Discussion

The main finding of Experiment 1 was the surprising cross-over interaction between Prime Type (Conceptual vs. Repetition) and Memory Judgment (R vs. K) on the priming effect (difference between matching and non-matching masked primes prior to test items in a recognition memory task): While Repetition primes increased K but not R judgments, replicating several previous studies (e.g., Rajaram, 1993; Woollams et al., 2008), Conceptual primes increased R but not K judgments. The latter finding for Conceptual primes would seem incompatible with previous accounts of masked priming of recognition test items, i.e., in terms of an increased fluency of processing test items being erroneously attributed to the past (Jacoby & Whitehouse, 1989), since this would seem to align more closely with the concepts of familiarity and K judgments, rather than the concepts of recollection and R judgments.

The pattern of Conceptual and Repetition priming effects was also different for Hits and False Alarms: Repetition priming increased K-Hits and K-FAs, whereas Conceptual priming increased R-Hits only. The same pattern was found in Taylor et al. (in press). This is further evidence that Conceptual and Repetition primes had qualitatively different effects (for example, in signal-detection terms, Conceptual primes may increase discriminability, i.e., shift only the distribution of studied items, whereas Repetition primes may affect response criterion, e.g., by shifting both studied and unstudied distributions with respect to the criterion). The selective increase of *correct* R responses caused by Conceptual priming is also difficult to reconcile with a fluency-attribution framework, which would predict increased “old” responses for more fluent (primed) items irrespective of Study Status (hence the label “memory illusion”).

These priming effects in terms of increases in the proportion of “old” judgments were mirrored by decreases in RTs, at least for Studied items. Decreased RTs following priming are expected if fluent processing induced by a matching prime affects the recognition judgment, i.e., speeding up “old” judgments to studied items, but slowing down “new” judgments to unstudied items (though the latter was not significant here; see also Taylor et al., in press). Although the same interactions with Prime Type (Conceptual vs. Repetition) were not found in RTs, as they were found in proportions of “old” judgments, the general pattern of RTs suggested that there was no obvious speed-accuracy trade-off entailed, at least for studied items.

We found no evidence that the interaction between Conceptual versus Repetition Primes and R versus K judgments differed according to either subjective or objective measures of participants’ ability to see the masked primes (even though the latter was above chance). While the critical factor(s) in masked priming of recognition test items remains unresolved – e.g., whether it is awareness (Jacoby & Whitehouse, 1989), attention (Klinger, 2001), a perceptual heuristic (Higham & Vokey, 2000) or prime duration (Huber, Clark, Curran, & Winkielman, 2008) – the present prime visibility analyses suggest at least that participants were not using explicit knowledge of the two different types of primes to guide their R/K judgments (e.g., in a strategic manner).

**Fig. 5:**
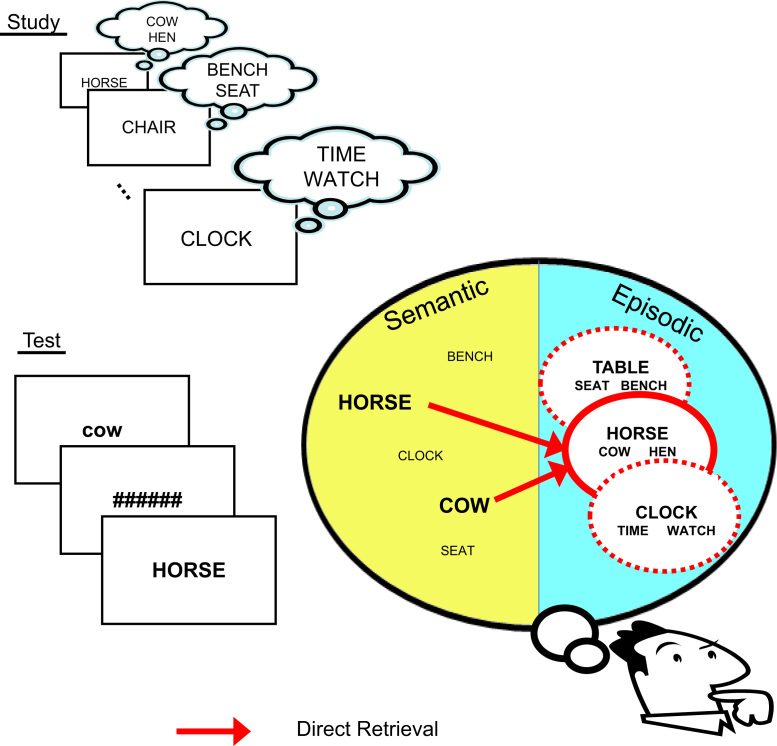
Schematic of how Conceptual primes might increase likelihood of direct episodic retrieval (recollection)—i.e., partial recollection of other information (internal source) generated at study, such as “Hen” (see text for further explanation).

How might the increased incidence of R judgments to studied items following conceptual primes be explained? Given evidence that semantically related concepts can be “activated” even when a prime is subliminal (Van den Bussche, Van den Noortgate, & Reynvoet, 2009), the “coming to mind” of such concepts (in the absence of knowledge of the source of that coming to mind) might be sufficient lead to an R judgment. If this were the sole cause however, then an increase in R judgements (following related vs. unrelated conceptual primes) would also be expected for unstudied items. To explain why the increase in R judgments occurred only for studied items, there must be some interaction between the (subliminal) activation of the prime concept and the memory trace laid down at Study. Given that the Study task entailed reasonably deep, elaborative processing (in order to judge a word's “interestingness”), it is likely that the concept denoted by the Conceptual prime at test was one of the concepts generated at study, at least on some trials, and therefore incorporated into an episodic trace. The combined activation of both the target and the prime at Test may therefore have increased the match with such episodic traces, relative to the unrelated (unprimed) case. According to the proposal in the Introduction however, such an increased “match” signal would be experienced as familiarity rather than recollection, so should increase K rather than R judgments. Therefore our preferred interpretation is that the activation of prime and target at Test actually increases the probability of retrieval of the complete episodic trace (i.e., combine to function as a more effective retrieval cue). This retrieval would then include additional information from the Study phase (such as other semantically-related concepts, or even some unique spatiotemporal tag), which defines recollection, and hence prompts the participant to give an R judgment. This “partial recollection” hypothesis is illustrated in Fig. 5. This hypothesis can also explain why, when we replicated this conceptual priming effect in an fMRI study (Taylor et al., in press), we found correlations across participants between the size of their conceptual priming effect on R judgments, and the size of the neural response in brain regions previously associated with recollection.

The increase in K judgments following Repetition primes, on the other hand, might be explained by a global match signal, in which the “faster” or “stronger” activation of the test item (following a matching prime) increases the size of that signal. This may not increase the probability of retrieving an episodic trace however, resulting in a lack of additional contextual information, and hence a K judgment. Though the match signal will be lower on average for Unstudied than Studied items, the increase following repetition primes will still produce occasions where the signal is strong enough to surpass the criterion for a K judgment, and produce a false alarm (e.g., in signal-detection terms, both old and new distributions would be shifted to the right relative to the K response criterion)—i.e., such an effect of Repetition primes would increase K judgments to both studied and unstudied items (unlike for Conceptual primes).

We do not know at what stage in word processing the repetition primes might boost activation (i.e., it could arise at one or more orthographic, phonological, lexical or even semantic stages). This then raises the question of why the semantic overlap entailed by repetition primes does not also increase the probability of retrieving an episodic trace in the manner described above for Conceptual primes. One possibility is that it is only the presence of additional, alternative concepts (activated by a conceptual but not repetition prime) that increases the probability of retrieving an episodic trace. One way to test this hypothesis might be to switch to a paired-associative cued-recall paradigm, in which unrelated pairs of words are studied, and then the single word used as the cue at test could be preceded by a third, masked prime word that was either semantically related or unrelated to the studied associate. If this conceptual prime increased the probability of recalling the associate, then this would be an objective indication of recollection (without requiring a subjective R judgment), which could not be explained by a generate-and-recognise strategy, given that the cue (test item) was unrelated, and that the prime, though related, is unlikely to be used in a conscious generation process (assuming that it was not consciously perceived).

Before switching to other paradigms like cued recall, however, we are faced with the puzzle that an increase in R judgments following semantically related primes was not found by Rajaram and Geraci (2000), who found an increase in K judgments instead, i.e., the same as when using repetition primes. One reason for this discrepancy may relate to the fact that their primes were also associatively related, rather than purely semantically related. This may have induced a different type of fluency (e.g., lexical), or may have partially activated associates at test that were different from those generated during the (semantic) study task (which may interact further with differences in the two study tasks: interestingness judgments in the present case versus intentional memorisation in Rajaram & Geraci, 2000). A related potential explanation comes from the definition of association, i.e., when one word is read, the other is likely to come to mind. On test trials with associative primes, participants may feel that the test cue made the prime word “come to mind”, which is not an unusual experience (by definition), and perhaps even an expected one, and therefore would not differentiate the Study episode from other prior experiences with the test cue. By contrast, on test trials with conceptually related (but non-associated) primes, the experience of the target bringing the prime to mind would (by definition) be a low-probability experience, and would therefore be diagnostic of the Study episode.

Another possible reason for the discrepancy across studies is that Rajaram and Geraci used unmasked primes (150 ms duration, followed by a 100 ms gap before the test item), which may affect, for example, how participants attribute fluency (Jacoby & Whitehouse, 1989), or at least, how they process the primes (Higham & Vokey, 2004; Klinger, 2001). As with the ‘definition of association’ argument above, the visibility of unmasked primes might allow participants to dismiss any retrieval of other associations also generated at Study as being due to the visible prime, and not diagnostic of memory for the Study episode. Yet the item-related fluency induced by such visible, associatively related primes may be more subtle and so less likely to be dismissed, and hence interpreted as familiarity, resulting in the increase in K responses found by Rajaram and Geraci.

Interestingly, two other recent studies have found priming effects on R responses: Kurilla and Westerman (2008) and Brown and Bodner (2011). They replicated the finding that masked repetition primes only affect K judgments under the standard (exclusive) R/K procedure, but found that they affected both R and K ratings when using the parallel ratings procedure of Higham and Vokey (2004), where a rating of 1–4 is made for both Remembering and Knowing of each test item. Whether parallel R/K ratings are justified theoretically, in terms of recollection and familiarity, depends on whether or not one regards these processes as mutually exclusive: If one can only ever recollect or experience familiarity (but not both; e.g., Gardiner & Parkin, 1990), then parallel R/K ratings might be difficult for participants to interpret; if on the other hand recollection and familiarity can co-occur (e.g., are either redundant or independent), then parallel R/K ratings would appear to provide a more direct measure than the traditional single judgment (e.g., in not requiring an adjustment of K proportions by R proportions to estimate familiarity, Yonelinas & Jacoby, 1995).

In the present context though, we consider a related but different possibility: That forcing participants to use only one of two response categories (in the standard R/K procedure) might obscure the underlying causes, to the extent that dissociations between R/K judgments that result may have little to do with recollection or familiarity. Participants in Rajaram and Geraci's study only experienced semantically related primes, whereas participants in Experiment 1 (and its replication in Taylor et al., in press) were exposed to both conceptual primes and repetition primes. Thus, even if participants were not aware of the primes (or at least, not aware of the two types of prime), they may have experienced qualitatively different effects of each type of prime, which they may have expressed differentially through R vs. K judgments. Therefore in Experiment 2a and 2b, we repeated the same basic procedure as Experiment 1, but using only conceptual primes.

## 4 Experiments 2a and 2b

Experiments 2a and 2b were two attempts to replicate the effect of conceptual primes on R judgments that was found in Experiment 1 (and in Taylor et al., in press), but without using repetition primes in the same experiment. Experiments 2a and 2b were identical, differing only in the participants tested.

### 4.1 Materials and methods

#### 4.1.1 Participants

Participants met the same criteria as Experiment 1. In Experiment 2a, a total of 25 participants (11 females) were recruited from the University of Cambridge, with a mean age of 21 (SD=2) years. In Experiment 2b, a total of 27 participants (17 females) were recruited from the MRC-CBU volunteer panel, with a mean age of 24 (*SD*=6) years.

#### 4.1.2 Materials and procedure

The stimuli and procedure were identical to those of Experiment 1, except that Conceptual primes (or Unrelated primes) were used in all four blocks; i.e., Repetition primes were never used. In the Prime Visibility Test, only two response options (candidate primes) were displayed, corresponding to the Conceptual Prime and the Unrelated Prime.

#### 4.1.3 Data analyses

The data were analysed as in Experiment 1, with the exception of the Priming Type (Conceptual, Repetition) factor, which no longer applied since all blocks were Conceptual Priming blocks.

### 4.2 Results (Experiment 2a)

#### 4.2.1 Proportion of trials

One participant was removed because their Pr score for Rs (.05) was three standard deviations smaller than the mean of the other participants (leaving 24 participants). The mean proportions of responses in each condition are shown in Table 3. For R judgments, overall accuracy (Pr) was .59, which was significantly greater than zero, *t*(23)=16.9, *p*<.001. For K judgments, accuracy was .39, which was also significantly greater than zero, *t*(23)=10.8, *p*<.001.^7^

**Supplementary Fig. 1:**
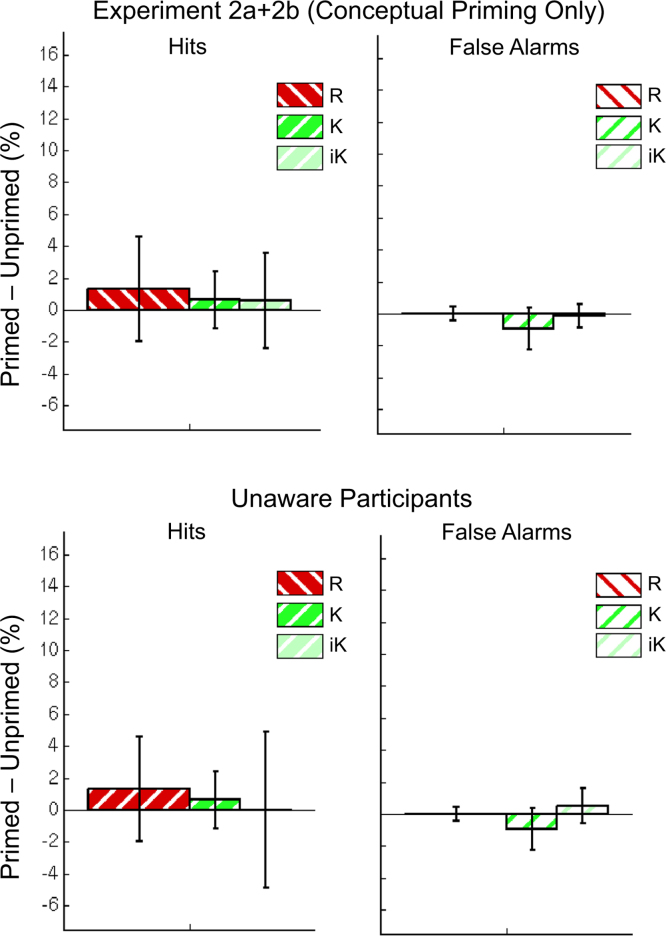


For “old” judgments, the 2 (Memory Judgment)×2 (Study Status)×2 (Prime Status) ANOVA revealed no main effects or interactions involving Prime Status—i.e., no priming effects (though the main effect of Study Status and the interaction between Memory Judgment and Study Status were both significant, *F*(1,23)s>25.6, *p*s<.001). Priming scores for each condition separately were not significant either for R or K judgments to either Studied or Unstudied items, *t*(23)s<1.25, *p*s>.22 (see Supplementary Fig. 1, top panel).

#### 4.2.2. Response time

As in Experiment 1, an ANOVA on median RTs for correct “old” and “new” decisions was performed (excluding one participant with insufficient trials; Table 4). No main effect or interaction involving Prime Status (i.e., priming effect) was significant.

#### 4.2.3. Prime visibility test

Thirteen of the 24 participants (54%) reported being aware that there were “hidden” words in the experiment; eight of these “aware” participants reported being able to identify prime words on some trials. In the Prime Visibility Test, mean performance was 79.0% (*SD*=15.8%), which was significantly better than chance (50%), *t*(23)=9.19, *p*<.001. Performance did not differ between Primed (*M*=78.6%, *SD*=19.8%) and Unprimed (*M*=79.3%, *SD*=16.2%) conditions (*t*s<1).

### 4.3 Results (Experiment 2b)

#### 4.3.1 Proportion of trials

The mean proportions of responses in each condition are shown in Table 3. For R judgments, overall accuracy (Pr) was .53, which was significantly greater than zero, *t*(26)=13.6, *p*<.001. For K judgments, accuracy was .36, which was also significantly greater than zero, *t*(26)=10.1, *p*<.001.^8^

For “old” judgments, the 2 (Memory Judgment)×2 (Study Status)×2 (Prime Status) ANOVA again revealed no main effects or interactions involving Prime Status—i.e., no priming effects. Priming scores for each condition separately were not significant either for R or K judgments to either Studied or Unstudied items, *t*(23)s<1.41, *p*s>.17 (see Supplementary Fig. 1, bottom panel).

#### 4.3.2. Response time

The ANOVA on median RTs (after removing one participant with <2 trials per cell; Table 4) did not reveal any significant main effect or interactions involving Prime Status (i.e., priming effects).

#### 4.3.3. Prime visibility test

Sixteen of the 27 participants (59%) reported being aware that there were “hidden” words in the experiment; eight of these “aware” participants reported being able to identify prime words on some trials. In the Prime Visibility Test, mean performance was 78.0% (*SD*=15.8%), which was significantly better than chance (50%), *t*(26)=9.08, *p*<.001. Performance did not differ between Primed (*M*=74.8%, *SD*=20.7%) and Unprimed (*M*=80.3%, *SD*=14.9%) conditions (*t*s<1).

### 4.4 Combined results (Experiments 2a+2b)

**Fig. 6:**
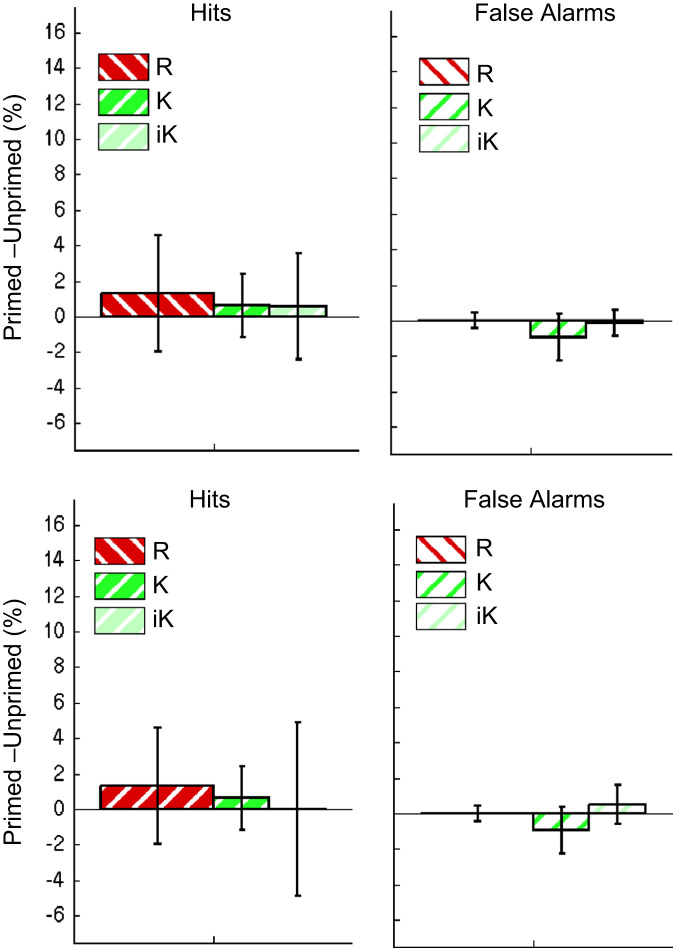
Priming (% of trials called “old” for Primed–Unprimed) as a function of Studied (Hits; left panels) or Unstudied (False Alarms; right panels) items, and separately for participants who reported being unaware of primes (lower panels), for R (red) vs. K (green) judgments when averaged across Experiments 2a and 2b. The two bars for K judgments are based on raw numbers (dark green) or under independent (IRK) scoring (‘iK’, light green; see main text). Note only Conceptual primes were used (cf. Experiment 1). Error bars are 95% confidence intervals.

For potential improved power, we averaged the priming scores across Experiments 2a and 2b. However, there were still no significant priming effects (Fig. 6, top panel). Based on the effect size from Experiment 1, a post hoc power analysis suggested that with the combined 51 participants, the power to detect the (one-tailed) increase in R Hits following conceptual primes was 89%. A further ANOVA with an additional factor distinguishing participant groups that did (*n*=30), or did not (*n*=21), subjectively report awareness of primes in Experiments 2a–2b again failed to reveal any significant effects involving Prime Status, *F*s<1. The same was true when analysing the “unaware” group separately, *F*s<1 (see Fig. 6, bottom panel) or under non-independent scoring of K.

## 5 Discussion

Experiment 1, and Taylor et al. (in press), had shown a surprising increase in R judgments (to studied items) following masked Conceptual primes, at least when the same participants also experienced the effects of masked Repetition primes in other blocks. Experiments 2a and 2b were attempts to replicate this effect, but only ever using Conceptual primes. Despite the fact that this entailed twice as many trials to estimate the effects of Conceptual primes (relative to Experiment 1), and twice as many participants (when collapsing across Experiments 2a and 2b), we failed to find any priming effects. These null results did not seem to depend on whether participants were aware of the presence of the masked primes. We consider possible explanations for this discrepancy between experiments in the General Discussion.

## 6 General discussion

The initial impetus of this paper was the unexpected finding in Experiment 1 that masked, semantically related (conceptual) primes increased participants’ tendency to indicate that studied items in a recognition task were “remembered” (i.e., given an “R” judgment; Tulving, 1985). This finding does not sit easily with current conceptions of recollection, given that previous interpretations of such masked priming of test items have been couched in terms of familiarity instead (e.g., Jacoby & Whitehouse, 1989; Rajaram, 1993; Rajaram & Geraci, 2000), and that familiarity has been most strongly tied to conceptual fluency (Yonelinas, 2002). In Section 3, we proposed one hypothetical interpretation, in terms of an increased probability of retrieving an episodic trace when there are multiple retrieval cues that overlap with semantically related concepts that were encoded together with the target item during study. This hypothesis is based on our present definition of recollection as a form of cued recall in which new information from episodic memory is retrieved, and assumes that participants use the R judgment appropriately in such cases. It is consistent with our findings that this conceptual priming of R judgments only occurs for Studied items (not Unstudied false alarms) and with our recent fMRI finding that activity in regions of the parietal cortex, which has been associated with recollection, correlate with the size of this conceptual-priming effect (Taylor et al., in press). It is also a productive hypothesis that can be tested in future, for example using cued recall (see Section 3).

However, the conceptual-priming effect was only found when conceptual primes occurred in the same experiment as did repetition primes (albeit in separate blocks): i.e., the same effect was not found in Experiment 2a/2b, where participants were only ever exposed to conceptual primes. We do not think the significant increase in R judgments to studied items following conceptual primes in Experiment 1 was a Type I error, because we replicated the same behavioural effect (and the interaction between Prime-Type, Memory Judgment and Prime Status) in an independent group of participants in Taylor et al. (in press). It is possible that the failures to replicate this effect in Experiments 2a and 2b were Type II errors, though again, a post hoc estimate showed that the combined data from both experiments had a reasonably high statistical power (89%) to detect such an effect. Therefore, we assume that the exposure to repetition primes (the only procedural difference of note between Experiment 1 and Experiments 2a and 2b) is a critical factor in explaining these data. This prompted us to reconsider the methodological issues surrounding estimating recollection within the recognition memory paradigm (as reviewed in Section 1.1), particularly in relation to the R/K procedure, as expanded below.

### 6.1 Methodological considerations

One possible explanation of the combined results of Experiments 1 and 2 is that, given only two response options following an old decision (i.e., R versus K), and given two qualitatively different experiences of fluency (i.e., following repetition versus conceptual primes), participants are forced to use one memory judgment for fluency of one type of processing, and the other judgment for fluency of another type of processing—resulting in the cross-over interaction between Priming Type and Memory Judgment that we found in Experiment 1 (and in Taylor et al., in press). In other words, this interaction may be an “artefact” of the binary (either–or) nature of the R/K procedure. In this case, one might not expect to find such a double dissociation if one used the parallel R–K ratings procedure of Higham and Vokey (2004; see also Brown & Bodner, 2011; Kurilla & Westerman, 2008), though even then, the restriction to only two types of ratings might favour the same type of dissociation. Other alternatives would be to allow a third option (e.g., of guesses; Tunney & Fernie, 2007, in case the present R–K dissociation could be simply a difference in recognition confidence following conceptual vs. repetition priming), or to allow indication of different types of recollection (e.g., of internal vs. external source), or possibly even to minimise recollection using the modified R/K procedure of Montaldi et al. (2006).

However, even this methodological interpretation, in terms of binary R/K categories, does not explain why participants happened to choose the R category for Conceptual primes and the K category for Repetition primes (or at least, why the majority of participants did so, such that the interaction between R/K and Priming Type was reliable over participants). Nor does it explain why the Conceptual Priming effect (in Experiment 1 and Taylor et al., in press) was restricted to Studied items, whereas the Repetition Priming effect on K judgments was found, and has previously been found, for both Studied items (Hits) and Unstudied items (False Alarms). Most importantly however, this methodological account does not explain why conceptual primes increased neither R nor K judgments significantly in Experiments 2a and 2b: even if the participants in these experiments did not experience the fluency induced by repetition primes, with which to contrast the fluency induced by conceptual primes, they should have shown an increase in one type of memory judgment (or at least on the number of “old” judgments overall, collapsing R and K judgments).

### 6.2 Theoretical considerations

A comprehensive explanation of the present results and those of Taylor et al. (in press) must therefore account for both the evidence that the Conceptual Priming effect appears to be a *bona fide* increase in recollection (rather than an artefact of the binary-response procedure used), and that this effect can disappear in certain stimulus list contexts (i.e., when only Conceptual and Unrelated primes are present). Effects of list context in priming are not uncommon: For example, in a lexical decision task with masked semantic primes, the ratio of related- to unrelated-prime trials modulates the size of RT priming effects, even when participants are unaware of the masked primes (Bodner & Masson, 2003).

List-context effects could arise by affecting how primes are processed, e.g., whether primes are passively ‘accepted’, attended, or actively inhibited, or by affecting decision processes, such as response criteria or, perhaps most pertinent to the present data, the *attribution* process. In the present study, two findings are consistent with the locus of the list-context effect being how primes are processed. First, in the Prime Visibility Test (Experiment 1), Conceptual (and Unrelated) Primes were more visible than Repetition primes (significantly so in Taylor et al., in press), suggesting that primes would have been *on average* more visible in Experiments 2a and 2b (Conceptual Priming only) than in Experiment 1 (Conceptual and Repetition Priming). Second, there were no priming effects on RT in Experiments 2a and 2b, suggesting that Conceptual Primes did not increase fluency of test-cue processing in those experiments. Taken together, these findings are consistent with participants in Experiments 2a and 2b (Conceptual priming only) being more likely to see primes, and perhaps finding the primes distracting or of no use, actively ignoring/inhibiting them, preventing priming of both RT and proportion of trials. However, it seems unlikely that a conscious strategy of inhibiting primes could fully account for the results of Experiments 2a and 2b since the subset of participants who reported being unaware of the masked primes still showed no evidence of Conceptual priming (and more generally, prime awareness has not interacted with priming effects in any of our experiments). Further, we note that null priming effects in RT are difficult to interpret in the present procedure because response accuracy is emphasised over speed.

An attribution account, on the other hand, does not require conscious awareness of primes; rather – as with the ‘binary-response artefact’ explanation considered in the previous section – it pertains to the participants’ experience of fluency as a consequence of priming. If such an attribution is indeed necessary for recollection to occur (or at least for a “Remember” judgment to be made), then the critical difference between Experiment 1 (and Taylor et al., in press) and Experiments 2a and 2b could be the likelihood of such an attribution occurring. In Experiments 2a and 2b, Conceptual primes occur on 50% of trials—twice as often as in Experiment 1 (and Taylor et al., in press). This higher proportion of conceptually primed trials might have caused participants to discount conceptual fluency as a memory signal, or in terms of the explanation proposed in the Section 3 (Fig. 5), it might have biased participants against using the retrieval of other, semantically related concepts as the basis for a ‘remember’ judgment. This account fits well with the notion that, at least in the case of familiarity, fluency must be *unexpectedly* high, *relative* to that of other items, in order for a (mis)attribution to memory to occur (e.g., Whittlesea & Williams, 1998, 2001). A challenge to this account is the finding that Repetition priming of K responses occurs when such primes occur on 50% of trials (e.g., Woollams et al., 2008); however, it is possible that the criteria for attributions of recollection and of familiarity are independent, and differentially influenced by parameters such as the proportion of related items.

The role of the attribution process in recollection has received little attention; however, Andrew Mayes and colleagues rather presciently raised this issue many years ago (Mayes, Gooding, & van Eijk, 1997). They hypothesised that one could easily confuse an imagined item-context association with a remembered one, and that “recollection must involve representing the item-context association and making an attribution that one is remembering.” Attributions of recollection might become particularly apparent when the “context” under consideration is internally generated (see discussion of internal vs. external source information in the Section 1.1), such as when participants engage in relatively deep semantic processing at Study and then use retrieval of semantically-related information at Test as a basis for “Remember” judgments. Note that the fluency that is attributed to remembering could still refer to the ease of retrieving new information (from multiple cues, as in Section 3), rather than the ease of processing an external stimulus (as is perhaps the case for attributing fluency to familiarity). Indeed, the fluency of retrieving new information in response to a cue may be a characteristic of episodic retrieval, to which we are finely tuned (spontaneous occurrence of which could be further used to trigger a retrieval mode; Tulving, 1983). These ideas clearly need further development. Nonetheless, this attribution account makes a prediction for future studies: The conceptual priming effect on “Remember” responses should be modulated by task demands that affect the attribution process (e.g., proportion of related items, instructions about primes, etc.), whereas concomitant priming effects on neural indices of recollection (e.g., activity in parietal cortex, amplitude of the late parietal ERP component; see, e.g., Taylor et al., in press) should not be so modulated (rather other regions/indices might be modulated, reflecting the attribution process).

### 6.3. Conclusion

Though we have not yet resolved the precise explanation (or boundary conditions) for conceptual primes to increase R judgments, the experiments reported here clearly illustrate the theoretical point made in Section 1.1 that estimating recollection and familiarity within the recognition memory paradigm is a difficult enterprise, which requires detailed task analysis and consideration of several methodological issues. Thus the application of “standard” paradigms (such as the R/K procedure), and even “standard” measurement models, such as the independent, dual-process model (e.g., Yonelinas, 2002), should be qualified by detailed task analyses, and ideally detailed process models (e.g., Norman, 2010); a message that we believe resonates with the continued work of Andrew Mayes and his colleagues.

## Figures and Tables

**Table 1:** Mean percentage of responses in Experiment 1 (with min–max range in brackets) to Studied or Unstudied words that were given each type of Memory Judgement (R, K, New) for each Prime Type (Conceptual/Repetition) and Prime Status (Primed/Unprimed), out of 60.

Memory judgement	Repetition primes				Conceptual primes			
	Studied		Unstudied		Studied		Unstudied	
	Primed	Unprimed	Primed	Unprimed	Primed	Unprimed	Primed	Unprimed
R	58.1 (17–88)	60.6 (12–90)	.6 (0–3)	.5 (0–3)	59.6 (28–90)	55.1 (6–83)	.5 (0–3)	.5 (0–3)
K	25.5 (3–53)	20.5 (0–50)	9.8 (0–28)	5.7 (0–22)	24.1 (1–52)	24.2 (5–47)	5.4 (0–18)	6.9 (0–30)
New	16.4 (3–48)	18.8 (5–50)	89.6 (68–100)	93.8 (78–100)	16.3 (0–43)	20.7 (3–50)	94.2 (82–100)	92.6 (70–100)

**Table 2:** Mean of median RT in Experiment 1 (with standard deviation in brackets) to Studied or Unstudied words that were given each type of Memory Judgement (R, K, New) for each for each Prime Type (Conceptual/Repetition) and Prime Status (Primed/Unprimed). Note:−=insufficient numbers to estimate RT (see Table 1).

Memory judgement	Repetition primes				Conceptual primes			
	Studied		Unstudied		Studied		Unstudied	
	Primed	Unprimed	Primed	Unprimed	Primed	Unprimed	Primed	Unprimed
R	805 (128)	843 (87)	–	–	837 (106)	857 (94)	–	–
K	983 (183)	1066 (193)	–	–	991 (159)	1006 (140)	–	–
New	–	–	873 (124)	837 (110)	–	–	851 (120)	868 (134)

**Table 3:** Mean percentage of responses in Experiments 2a and 2b (with min–max range in brackets) to Studied or Unstudied words that were given each type of Memory Judgement (R, K, New) for each Prime Status (Primed/Unprimed), out of 120.

Memory judgement	Experiment 2a (Conceptual)				Experiment 2b (conceptual)			
	Studied		Unstudied		Studied		Unstudied	
	Primed	Unprimed	Primed	Unprimed	Primed	Unprimed	Primed	Unprimed
R	61.1 (27–91)	59.8 (22–90)	1.0 (0–6)	1.0 (0–7)	54.1 (14–96)	54.4 (13–91)	.8 (0–5)	.5 (0–3)
K	17.9 (3–33)	17.3 (0–33)	7.8 (1–30)	8.8 (0–29)	18.2 (0–42)	18.7 (2–48)	6.3 (0–21)	5.7 (0–21)
New	20.9 (6–63)	22.7 (2–67)	91.2 (64–99)	90.2 (64–100)	27.8 (2–86)	27.0 (3–85)	93.0 (78–99)	93.9 (79–100)

**Table 4:** Mean of median RT in Experiments 2a and 2b (with standard deviation in brackets) to Studied or Unstudied words that were given each type of Memory Judgement (R, K, New) for each Prime Status (Primed/Unprimed). -=insufficient numbers to estimate RT (see Table 1).

Memory judgement	Experiment 2a (Conceptual)				Experiment 2b (Conceptual)			
	Studied		Unstudied		Studied		Unstudied	
	Primed	Unprimed	Primed	Unprimed	Primed	Unprimed	Primed	Unprimed
R	825 (140)	822 (132)	–	–	874 (135)	876 (120)	–	–
K	1032 (185)	1010 (218)	–	–	1099 (182)	1087 (213)	–	–
New	–	–	851 (140)	860 (151)	–	–	863 (132)	870 (134)

## Footnotes

1 Particularly relevant here is the small set of response options, cf. the open-ended response set in recall paradigms. Open-ended response sets are more prone to strategic/executive confounds, which may be less available to patients, more difficult to elicit from nonhuman animals, and normally require spoken or written output, which tend to introduce movement-related artefacts in many neuroimaging techniques (though none of these issues is necessarily insurmountable).

2 One point on which our current conception of recollection might differ from that of Montaldi and Mayes (2010) relates to their statement: “[…] if context is the often repeatedly encountered situational background within which many different items are experienced, then it is not helpful to describe recollection as recall of context. Although one might recollect a study context when it is no longer present, the power of recollection as a recognition diagnostic aid depends primarily on the recall of unique item associates that occurred within the original study context” (p. 1295). We do not see such uniqueness as a defining feature of recollection. For example, if a test item brings to mind a detail about the room in which items were studied (and this detail was not already in mind at test, e.g., by virtue of being perceived, if the test phase is occurring in the same room, or by virtue of being retrieved from episodic memory following previous test items), then we would call this recollection, even if that detail had been associated with multiple items at study.

3 Under non-independence scoring (raw numbers of K judgments), these Pr values were .18 in Conceptual Priming and .15 in Repetition Priming blocks, both of which were again significantly greater than zero, *t*(25)s>5.0, *p*s<.001. The same ANOVA model reported in the main text but using raw K judgments also produced a 3-way interaction between Priming Type, Memory Judgement, and Prime Status, *F*(1,25)=6.59, *p*<.05, and an additional 3-way interaction between Priming Type, Study Status, and Prime Status, *F*(1,25)=12.9, *p*=.001.

4 The pattern of results was the same when raw K judgments (non-independence assumptions) were used: Priming Type×Memory Judgment×Prime Status interaction, *F*(1,46)=11.9, *p*<.001; Priming Type×Study Status×Prime Status interaction, *F*(1,46)=31.2, *p*<.001; interactions with awareness interactions: *F*s<1.48, *p*s>.23.

5 The pattern of results was the same under non-independence assumptions: For Unaware participants, Priming Type×Memory Judgment ×Prime Status *F*(1,22)=4.76, *p*<.05; Priming Type×Study Status×Prime Status, *F*(1,22)=18.1, *p*<.001.

6 For example, even the *n*=23 subjectively “unaware” participants performed above chance on the objective prime visibility test (*M*=61%, SD=26%, *t*(22)=5.10, *p*<.001), demonstrating that the ability to see the primes when alerted to their presence (in an objective test) is not necessarily a good indicator of self-reported awareness during the experiment itself.

7 For non-independence K, Pr=.09, which was significantly greater than zero: *t*(23)=4.9, *p*<.001. The main ANOVA on proportion of responses revealed significant main effects of Memory Judgment and of Study Status, and a significant interaction between Memory Judgment and Study Status, *F*(1,23)s>56.8, *p*s<.001; but as in the independence-K analysis, no effects involving Prime Status were significant.

8 For non-independent K, Pr=.12, which was significantly greater than zero, *t*(26)=5.38, *p*<.001. The ANOVA on proportion of responses produced no effects involving Prime Status.
